# Estimated Glomerular Filtration Rate and the Risk of Major Vascular Events and All-Cause Mortality: A Meta-Analysis

**DOI:** 10.1371/journal.pone.0025920

**Published:** 2011-10-19

**Authors:** Marion Mafham, Jonathan Emberson, Martin J. Landray, Chi-Pang Wen, Colin Baigent

**Affiliations:** 1 Clinical Trial Service Unit and Epidemiological Studies Unit (CTSU), University of Oxford, Oxford, United Kingdom; 2 Center for Health Policy Research and Development, National Health Research Institutes, Zhunan, Taiwan; University of Modena and Reggio Emilia, Italy

## Abstract

**Background:**

Lower estimated glomerular filtration rate (eGFR) has been associated with an increased risk of major vascular events (MVEs) and death, but differences in methodology make between-study comparisons difficult. We used a novel method to summarise the published results.

**Methods and Findings:**

Studies assessing the relationship between baseline eGFR and subsequent MVEs or all cause mortality were identified using Pubmed. Those which involved at least 500 individuals, planned at least 1 year of follow-up, reported age and sex adjusted relative risks, and provided the mean eGFR in each category (or sufficient information to allow its estimation) were included. To take account of differences in underlying risk between studies, proportional within-study differences in eGFR (rather than absolute eGFR values) were related to risk. Fifty studies (2 million participants) assessing MVEs and 67 studies (5 million participants) assessing all cause mortality were eligible. There was an inverse relationship between lower eGFR and the risk of MVEs and of death. In studies among people without prior vascular disease, a 30% lower eGFR level was on average associated with a 29% (SE 0.2%) increase in the risk of a MVE and a 31% (SE 0.2%) increase in the risk of death from any cause. In studies among people with prior vascular disease, these estimates were 26% (SE 1.0%) and 23% (SE 0.2%) respectively. While there was substantial statistical heterogeneity between the results of individual studies, a 30% lower eGFR was consistently associated with a 20-30% higher risk of both outcomes, irrespective of prior history of vascular disease or study design.

**Conclusions:**

Lower eGFR was consistently associated with a moderate increase in the risk of death and MVEs. If these relationships are causal and continuous, then around one fifth of vascular events among those over 70 years might be attributable to renal impairment.

## Introduction

Individuals requiring dialysis treatment have a 10 to 100 fold increased risk of vascular death compared with the general population [1], but represent less than 0.2% of the population [2]. In contrast, mild-to-moderate reductions in estimated glomerular filtration rate (eGFR) are common, especially among older people. In the United States, for example, only about 2% of people aged 40-59 years have an eGFR <60 ml/min/1.73 m^2^ but this proportion increases to about 25% among those aged over 70 years [3]. Several prospective cohort studies have suggested that such mild-to-moderate reductions in eGFR may be associated with a moderate, but clinically important, increase in the risk of major vascular events [4]–[7] and also of the overall risk of death [8], but this has not been a consistent finding in all populations studied [9]-[14].

We sought to perform a meta-analysis of the observational relationship between eGFR and the risk of major vascular events and mortality. In order to provide a meta-analytic summary of these relationships from studies with reference and disease groups which differed widely in their chosen cut points of eGFR, we developed a novel technique in which the published measures of relative effect in each study, adjusted as completely as possible for confounding, were related to the corresponding relative differences in mean eGFR between the disease and reference groups. This method minimises any biases resulting from variations in the creatinine assay [15] or the use of different equations to estimate GFR, because such biases ‘drop out’ of the mathematical calculations involved in relative comparisons.

## Methods

### Data sources and searches

Studies that had reported relationships between estimated glomerular filtration rate (eGFR), and major vascular events (MVEs), mortality, or both, were identified with a Pubmed search (1966 to 1^st^ September 2008). We used a combination of terms relating to vascular disease or death, creatinine or eGFR, and cohort studies (Appendix S1), and supplemented this electronic search with review of reference lists from subsequently retrieved papers and, where appropriate, contact with study authors. Only 3 studies [17]–[19] reported vascular and non-vascular mortality separately, so analyses are limited to assessing associations between eGFR and all-cause mortality. Studies were included if eGFR was estimated by the Modification of Diet in Renal Disease (MDRD) study [20] or the Cockcroft-Gault [21] formulae. For each study we aimed to identify a composite outcome, MVE, that involved a combination of one or more of: myocardial infarction, unstable angina, stroke, transient ischaemic attack, coronary or non coronary revascularisation, or vascular, cardiac or coronary death.

### Study selection

Studies involving at least 500 individuals and 1 year of follow-up were eligible if they reported an age- and sex-adjusted association between eGFR and all-cause mortality, eGFR and MVEs, or both. Studies were included if they were conducted among apparently healthy individuals, among patients with known prior vascular disease or among individuals with an increased risk of vascular disease (e.g. patients with hypertension). Studies in populations with pre-existing chronic kidney disease (CKD) or serious non-vascular disease were excluded. We included studies only if the publication reported a relevant association in terms of a comparison between two or more categories of individuals defined by eGFR cut-points (eg, a relative risk corresponding to comparison between those with eGFR <60ml/min/1.73m^2^ and those with eGFR ≥60ml/min/1.73m^2^).

### Data extraction and quality assessment

We extracted, from each study, details of the study population, mean follow-up, the type and total number of outcomes, the cut points used, the mean eGFR, the number of individuals and outcomes in each eGFR category, confounding variables for which adjustment had been made in the most complete regression model, and relative risks and confidence intervals for each relevant comparison under that model. A non-randomised study was classified as a “prospective cohort study” if the baseline data were collected prospectively, with contemporaneous assessment of clinical measurements and laboratory blood tests using standardised methods. In most of these studies, participants were actively followed up at study visits or by telephone or postal questionnaire with subsequent confirmation of major vascular events using hospital records. A study which extracted data from health care records retrospectively was classified as a “retrospective cohort study”. If separate reports were available from a single study population, the first published paper was used unless a subsequent report contained additional events. In studies where “pooled” analyses had been performed in the original papers [6]; [22]; [23], authors were asked to provide study-specific results. In one major study in which age-specific analyses were performed, the authors were contacted to provide separate age-specific and overall results for people with or without prior vascular disease [8]. In another study, in which the disease and reference groups were defined by both eGFR cut-points and the presence and absence of proteinuria, the authors were contacted to provide results based on cut points of eGFR alone [19].

### Data synthesis and analysis

The primary analyses involved estimating the relative risk of a MVE and of all-cause mortality associated with a 30% lower eGFR. (Among 20536 participants in the MRC/BHF Heart Protection Study [24], this was the approximate proportional difference in mean baseline eGFR between those with an eGFR of 60–89 ml/min/1.73 m^2^ [mean eGFR 74 ml/min/1.73 m^2^] and ≥90 ml/min/1.73m^2^ [mean eGFR 101 ml/min/1.73 m^2^], and between those with an eGFR 30–59 ml/min/1.73m^2^ [mean eGFR 51 ml/min/1.73m^2^] and 60–89 ml/min/1.73 m^2^). The results for individual studies are presented with 99% confidence intervals, whilst summary results are presented with 95% confidence intervals. In order to explore the possible role of reverse causality (ie, a history of vascular disease leading both to an increase in risk and a reduction in eGFR), we subdivided studies into those in populations with known vascular disease and those which did not select individuals on the basis of prior vascular disease (ie, studies which either excluded those with prior vascular disease or consisted of unselected samples of the general population, the elderly or individuals with diabetes or hypertension). We analysed prospective cohort studies, retrospective cohort studies and randomised trials separately. To further assess the possible bias introduces by reverse-causality we also performed sensitivity analyses excluding studies that included individuals with an acute illness requiring hospitalisation at baseline.

### Calculating summary risk estimates for each study

Depending on the format of reporting in each study publication, study-specific relative risks corresponding to a 30% lower eGFR were estimated because such a decrement in eGFR is approximately equivalent to the differences between an eGFR of 90 and 60 ml/min/1.73 m^2^ or between 60 and 45 ml/min/1.73 m^2^, both of which correspond to cut-offs for KDOQI CKD stages. The relative risks for MVEs and for all-cause mortality were calculated using one of the following methods:

- Comparisons between two categories: the relative risk per 30% lower eGFR (RR*) was estimated from the published relative risk (RR) through the equation RR*  =  0.7^ln(RR)/ln(a/b)^, where b is the mean eGFR level in the reference group and a is the mean eGFR level in the single comparison group.
- Comparisons between more than two categories: logistic regression based on the number of subjects and events observed in each exposure group was used to estimate the variance-covariance matrix of the crude log odds ratios, which was then used to estimate the approximate “floated” standard error of the log odds ratio for each group (including the reference group) [25]. For each outcome, the relative risk associated with a 30% lower eGFR was estimated from the slope provided by the weighted linear regression of the *published* log relative risks on the (log) mean eGFR levels (with weights equal to the reciprocal of the square of the floated standard errors, ie, inverse variance weighted) with the standard error of the slope corrected by dividing it by the square root of the mean squared error (which is needed when regression weights are known exactly rather than just relatively) [26].

In studies that did not report mean eGFR levels for each analysis category we assumed a normal distribution for eGFR (since this was approximately the distribution observed in the Heart Protection Study [24]; data not shown) and calculated means using the conditional probability density function with the population mean and variance (Appendix S2). In one study [27], a log-normal distribution was assumed rather than a normal distribution because the authors explicitly stated that the distribution was positively skewed (the mean values in this study therefore represent geometric means). If the mean eGFR or standard deviation in the overall population was not reported, it was estimated from the proportions of the population falling within each group (Appendix S2). (The validity of this method was confirmed by using it to compare estimated and observed eGFR levels in the 38 studies that did report mean eGFR level in each group [Figure S1].)

### Assessing heterogeneity between studies

Given the summary log relative risk b_i_ (and its variance v_i_) for each study (see above), heterogeneity between the different studies was assessed by calculating ∑(w_i_b_i_^2^) - ∑ (w_i_b_i_)^2^/∑w_i_ (where w_i_  =  1/v_i_), and testing this against a chi-squared distribution with degrees of freedom equal to one less than the number of studies. The “pooled” log relative risk across different studies was calculated by ∑w_i_b_i_/∑w_i_ (with variance equal to 1/∑w_i_).

### Calculating the hypothetical population attributable risk fraction associated with reduced eGFR

In order to assess the potential impact of reduced eGFR on major vascular events within the population, we calculated hypothetical population attributable risk fractions (PARF) for three categories of reduced eGFR (60-89 ml/min/1.73m^2^, 30–59 ml/min/1.73 m^2^ and 15–29 ml/min/1.73 m^2^) using age specific prevalence estimates from the National Health and Nutrition Estimation Survey (1999 to 2000) [3]. The PARF for the jth eGFR category (j = 1,2,3) is given by p_j_(RR_j_ – 1)/(1 + ∑p_i_(RR_i_-1)) where p_j_ is the proportion of the population falling into the jth eGFR category and RR_j_ is the relative risk for the jth eGFR category compared with the reference group (eGFR ≥90 ml/min/1.73 m^2^). The overall PARF associated with reduced eGFR (<90 ml/min/1.73 m^2^) is then calculated by the sum of the PARFs for each eGFR category.

This study was conducted at the Clinical Trial Service Unit, University of Oxford and required no external funding.

## Results

**Figure 1:**
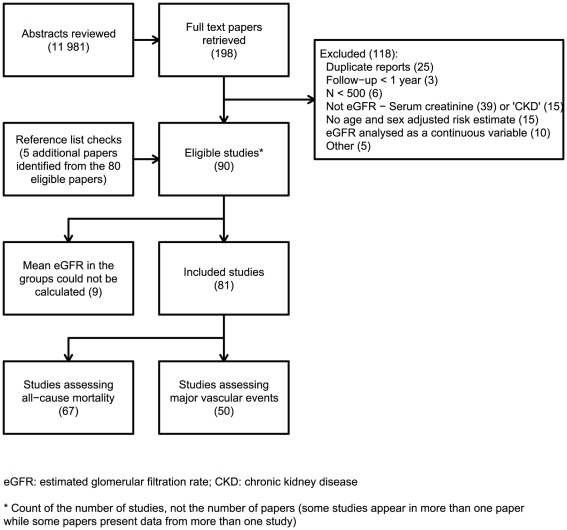
Results of the literature search.

Figure 1 summarizes the search retrieval process. Out of 11981 abstracts reviewed, 198 papers were retrieved for further examination, of which 80 met the inclusion criteria, with 5 more being identified from the reference lists. Contained within these 85 manuscripts was information relating to 90 different studies. Mean eGFRs in different risk categories were presented (or sufficient information was provided to allow their estimation) in 81 of these studies (58 cohort studies [28 prospective and 30 retrospective] and 23 randomised controlled trials (Table S1).

**Figure 2:**
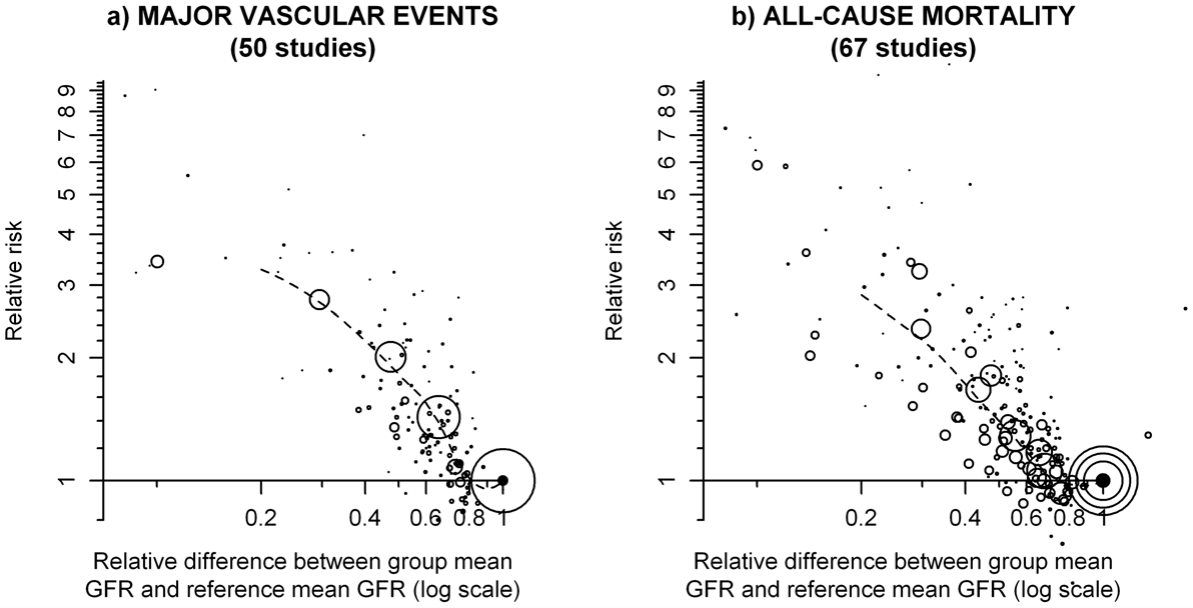
Relationship between eGFR and risk of major vascular events and all-cause mortality. Relative risks are shown on the log scale. The area of each plotting symbol is proportional to the amount of statistical information (i.e. it is inversely proportional to the variance of the floated log odds ratio). The dashed lines represent the best local polynomial regression fits.

Fifty studies (25 prospective cohort studies, 10 retrospective cohort studies and 15 trials) comprising a total of over 2 million individuals had assessed the relationship between eGFR and the risk of a major vascular event (MVE). The weighted mean (SD) eGFR in the studies' reference groups (1.6 million individuals, 78% of the sample) was 85 ml/min/1.73 m^2^ (14 ml/min/1.73 m^2^). A graded relationship was observed across the different studies with lower eGFR levels consistently related to higher MVE risk, at least down to about 25–30 ml/min/1.73m^2^ (Figure 2).

**Figure 3:**
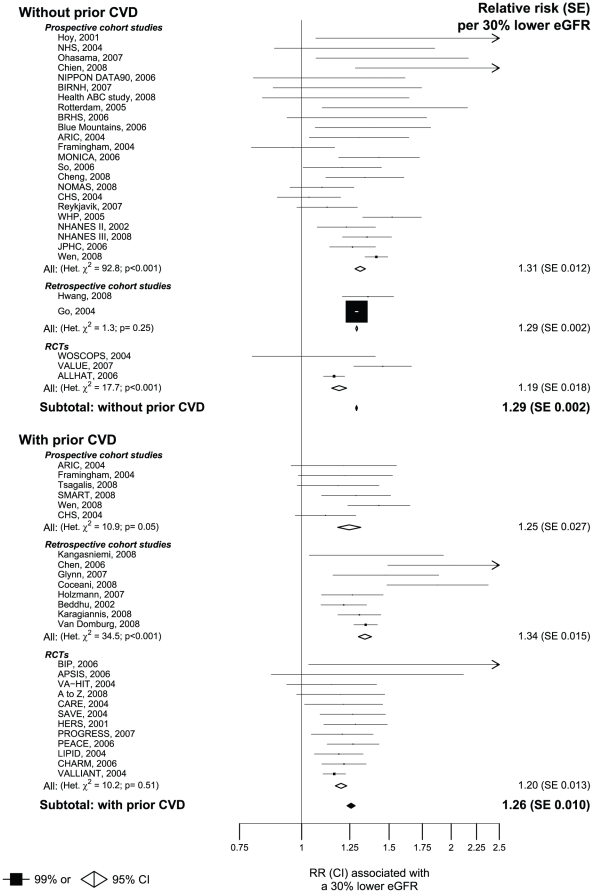
Meta-analysis of the association between eGFR and major vascular events.

In studies among people without prior vascular disease, each 30% lower eGFR level was associated with a 29% increase in the risk of a MVE (RR 1.29 [95% CI 1.28 to 1.30]: Figure 3). Similar estimates were obtained from the prospective cohort studies (RR 1.31 [95% CI 1.28 to 1.34]) and retrospective cohort studies (RR1.29 [95% CI 1.29 to 1.30]) but the randomised controlled trials yielded a slightly lower relative risk per 30% lower eGFR (RR 1.19 [95%CI 1.15 to 1.23]). There was substantial heterogeneity between the results of the different prospective cohort studies and randomised trials (Figure 3). The relative strength of the relationship between lower eGFR and risk of a MVE was similar among people with prior vascular disease (RR 1.26 [95% CI 1.23 to 1.28] per 30% lower eGFR: Figure 3). As in populations without vascular disease, the results were not substantially different in the different types of study examined (RR per 30% lower eGFR: 1.25 [95% CI 1.18 to 1.32] in prospective cohort studies, 1.34 [95% CI 1.30 to 1.38] in retrospective cohort studies and 1.20 [95% CI 1.17 to 1.23] in randomised controlled trials: Figure 3). There was significant heterogeneity between the results of the individual prospective cohort studies and individual retrospective cohort studies. In contrast, there was no significant heterogeneity between the results of the different randomised trials (Figure 3). Eight of the 26 studies assessing MVEs among people with known prior vascular disease included individuals with an acute illness requiring hospitalisation at baseline (Table S1)). Excluding these studies did not materially alter the results (RR per 30% lower eGFR 1.28 [95% CI 1.25–1.31]).

Sixty seven studies (19 prospective cohort studies, 31 retrospective cohort studies and 17 trials) comprising a total of 4.9 million individuals assessed the relationship between eGFR and all-cause mortality. Among these studies, 3.7 million individuals (76% of all participants) were included in the reference groups, in which the weighted mean (SD) eGFR across the studies was 85 ml/min/1.73 m^2^ (14 ml/min/1.73 m^2^). As was the case in analyses of MVEs, a graded relationship was observed between lower eGFR levels and progressively higher all-cause mortality risks (Figure 2b).

**Figure 4:**
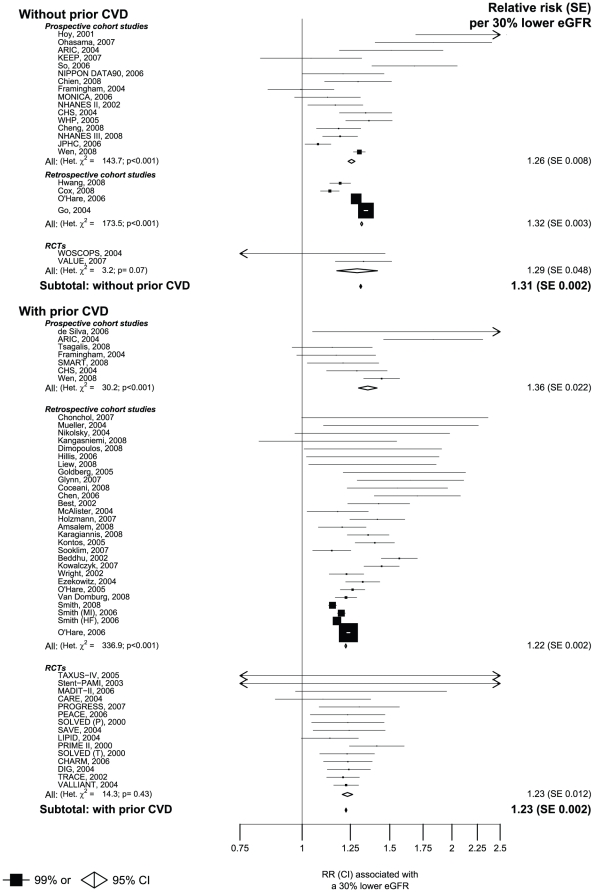
Meta-analysis of the association between eGFR and all-cause mortality.

In studies among people without prior vascular disease, a 30% lower eGFR level was, on average across the range studied, associated with a 31% increase in the risk of death from any cause (RR 1.31 [95% CI 1.31 to 1.32: Figure 4). The estimated RR per 30% lower eGFR was comparable across the different study designs (1.26 [95% CI 1.24 to 1.28] in prospective cohort studies, 1.32 [95% CI 1.31 to 1.32] in retrospective cohort studies and 1.29 [95% CI 1.17 to 1.42] in randomised controlled trials). Within the prospective and retrospective cohort studies, however, there was substantial heterogeneity between the results from the individual studies (Figure 4). In studies of individuals with prior vascular disease a 30% lower eGFR was associated with 23% increase in the risk of death from any cause (RR 1.23 [95% CI 1.22 to 1.23]: figure 4). A slightly higher relative risk was observed in prospective cohort studies (1.36 [95% CI 1.30 to 1.41]) than in the retrospective cohort studies (1.22 [95% CI 1.22 to 1.23]) or the randomised controlled trials (1.23 [95% CI 1.20 to 1.26]). Substantial heterogeneity was observed between the results from the different prospective and retrospective cohort studies, but not between the results from the trials (Figure 4). Among the 50 studies assessing all cause mortality in people with prior vascular disease, 24 included individuals who were acutely unwell at baseline (Table S1). Results were similar when these studies were excluded (RR per 30% lower eGFR 1.24 [95 CI 1.24–1.25]).

## Discussion

A large number of population-based studies have reported associations between estimated GFR and particular outcomes, but they have employed a wide variety of methods making it difficult to summarise the results of such studies quantitatively. In this meta-analysis, we have used a novel method of statistical synthesis and have shown that there are inverse relationships between proportional differences in baseline eGFR and the risks of all cause mortality and of major vascular events. Although there is substantial heterogeneity among the different studies, overall the studies indicate that a 30% lower eGFR was associated with approximately 20–30% greater risk of each outcome. The strength of the associations, as estimated by relative risks, did not appear to be influenced strongly by whether individuals already had a history of vascular disease, suggesting that these associations are unlikely to be attributable to reverse causality (whereby a history of vascular disease leads both to reduced eGFR and a higher risk of recurrence). Although we could not assess the relationship between eGFR and mortality at different ages using the pooled data, age-specific results were made available by the authors of one very large study of eGFR and all-cause mortality (the Veterans Affairs Study;[8]). The relative strength of the relationship between eGFR and all-cause mortality decreased with older age. For example, among those without prior vascular disease, the RR (95% CI) of death associated with a 30% lower eGFR was 1.43 (1.39 to 1.46) at ages 45-54 and 1.24 (1.22 to 1.27) at ages 75–84 (Figure S2). However, since older people have a higher annual risk of death, the absolute increase in deaths associated with lower eGFR is substantially greater among the elderly: for example, among people aged 45-54, a 30% lower eGFR was associated with about 50 extra deaths per 10 000 people per year as compared to about 100 extra deaths per 10 000 people per year among people aged 75–84. Similarly the absolute relevance of eGFR to all-cause mortality risk was greater in people with prior CVD.

In weighing the potential importance of these findings, several sources of bias need to be considered: (i) bias due to the limitations of data extracted from published data; (ii) bias due to methods of estimated GFR; and (iii) bias due to regression dilution. These biases are possible explanations for between-study heterogeneity in relative risk estimates, but also have the potential to influence the shape and strength of the overall associations observed.

Although our methods were designed to minimise the errors introduced by differences between studies in laboratory calibration, or in the particular statistical measures of association that were reported, there are nevertheless several other ways in which the use of summary data from published reports might introduce bias. For example, there was little uniformity in the definition of MVEs, with some studies considering only myocardial infarction, some just stroke, and others a composite of several types of vascular event (Table S1). Such variations could have resulted in heterogeneity if, as is plausible, the strengths of any associations with eGFR vary between different vascular outcomes. Similarly, since only 3 studies assessed vascular and non-vascular mortality separately [17]–[19], our analysis examined only all-cause mortality. Consequently, variation between studies in the proportions of deaths attributable to vascular disease could well result in heterogeneity in associations between eGFR and death from any cause. Furthermore, there were substantial differences between the studies in the extent to which adjustment was made for confounding. In many of the studies, particularly the retrospective cohorts, adjustment was made only for the presence of co-morbid disease abstracted from health care records, which might have led to an overestimation of the risks of MVE and death associated with lower eGFR because of residual confounding. However, some of the studies might equally have underestimated the relevance of eGFR by adjusting for factors, such as blood pressure, which are likely to be part of the mechanism by which reduced GFR might increase the risk of vascular disease and death, (ie, the “causal pathway”).

All of the studies in this meta-analysis used a creatinine-based method for estimating GFR. Other studies have shown that eGFR estimates are only weakly related to true GFR among individuals with eGFR in excess of about 60 ml/min/1.73 m^2^, so above this level there is likely to be misclassification of individuals between comparison and reference groups [28]. The anticipated effect of this would be to flatten the dose response curve among those with higher eGFR and would also result in underestimation of the strength of the relationships between true GFR and risk of vascular disease and all-cause mortality [29].

Regression dilution bias may also have resulted in distortion of the true dose-response risk-relationships [30]. To investigate this, eGFR estimates over about 5 years were examined among 7697 individuals allocated placebo in the MRC/BHF Heart Protection Study (HPS) [24]. Among individuals with baseline eGFR below 70 ml/min/1.73 m^2^, no regression to the mean upon re-measurement was observed (slope of follow-up log eGFR regressed on baseline log eGFR  =  1.06 [Figure S3]), whereas substantial regression to the mean was observed (slope = 0.65) among those with higher eGFR at baseline. Considered together, the non-uniform effect of correction for regression to the mean might be to straighten somewhat the inflection that is suggested among those with higher levels of eGFR in Figure 2.

Until recently, attempts to summarise the available data assessing the relationships between eGFR and important outcomes have been only semi-quantitive [16], [31]. However, a collaborative meta-analysis of 21 general population studies that were able to provide detailed individual data, published in June 2010 by the Chronic Kidney Disease Prognosis Consortium, reported relations between predefined categories of eGFR and albuminuria and 45 584 deaths from any cause and 9637 deaths due to cardiovascular disease [32]. Lower eGFR and increasing level of albuminuria were each independently associated with an increase in the risk of both outcomes [32]. However, among those with preserved eGFR (i.e. eGFR >60 ml/min/1.73 m^2^), the relationship between eGFR and these outcomes was flat, possibly – as described above – due to the weak relationship between creatinine and true GFR, and regression dilution bias. Since the CKD prognosis consortium included only 5 of the general population studies included in our meta-analysis, our results complement these findings by demonstrating the consistency of the associations across a large number of studies including populations with and without prior vascular disease.

**Figure 5:**
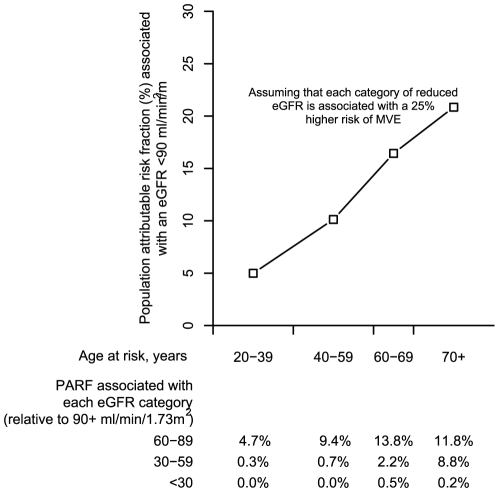
Hypothetical age-specific estimates of the population attributable risk fraction for major vascular events associated with reduced eGFR.

The present study cannot determine whether any of the observed associations are causal, although an association between reduced GFR and vascular disease is suggested by studies indicating that minor degrees of renal impairment following kidney donation result in permanent increases in blood pressure [33]; [34]. If it is assumed that the relationships are causal, how large might the contribution of reduced renal function to the risk of vascular disease in the general population be? In order to assess this hypothetically, prevalence data from the National Health and Nutrition Estimation Survey [3] were used to calculate population attributable risk fractions (PARFs) of MVEs for three categories of reduced eGFR (60–89 ml/min/1.73 m^2^, 30–59 ml/min/1.73 m^2^ and 15–29 ml/min/1.73 m^2^) in both middle and old age (ie the proportion of vascular events that would have been avoided if the risk among those with reduced eGFR was the same as among those with an eGFR ≥90 ml/min/1.73 m^2^). Figure 5 indicates that if each category of reduced eGFR was causally associated with about a 25% increase in the risk of a MVE, the combined PARF associated with an eGFR <90 ml/min/1.73 m^2^ would be about 10% among those aged 40–59 years rising to over 20% among those aged over 70 years. Since the incidence of vascular events is much higher among older people, the absolute number of excess vascular events potentially attributable to renal impairment would be substantially higher at older ages. Based on US death rates in 2005, we might expect 10 excess vascular deaths per 100,000 people per year among those aged 45–54 years compared to 400 excess vascular deaths per 100,000 people per year among those aged 75–84 years.

This meta-analysis suggests that a 30% lower eGFR is associated with a 20–30% increase in the risk both of major vascular events and of death from any cause which, if causal, would imply that up to 10% of vascular events in middle age and 20% in old age might be attributable to age-related changes in renal function. Given the potential size of the contribution of age-related loss of renal function to public health, the many uncertainties about the nature and strength of relationships between eGFR and individual vascular outcomes and cause-specific mortality in various populations requires further study.

## Supporting Information

Figure S1 **Comparison of actual vs estimated mean eGFR levels.**
(PDF)

Figure S2 **Age-specific association between eGFR and all-cause mortality in the Veterans Affairs Study.**
(PDF)

Figure S3 **Mean follow-up eGFR level by percentile of the baseline distribution among 7697 placebo patients in the Heart Protection Study (follow-up 4-5 years later).**
(PDF)

Table S1 **Characteristics of included studies.**
(PDF)

Appendix S1 **PubMed search conducted on 1st September 2008.**
(PDF)

Appendix S2 **Statistical appendix.**
(PDF)
