# Supplementary figures and images for: Estimated Glomerular Filtration Rate and the Risk of Major Vascular Events and All-Cause Mortality: A Meta-Analysis
Source: PLoS One. 2011 Oct 19;6(10):e25920. doi: 10.1371/journal.pone.0025920 (PMC3198450; doi:10.1371/journal.pone.0025920)

**FIGURE S1: COMPARISON OF ACTUAL VS ESTIMATED MEAN eGFR LEVELS**

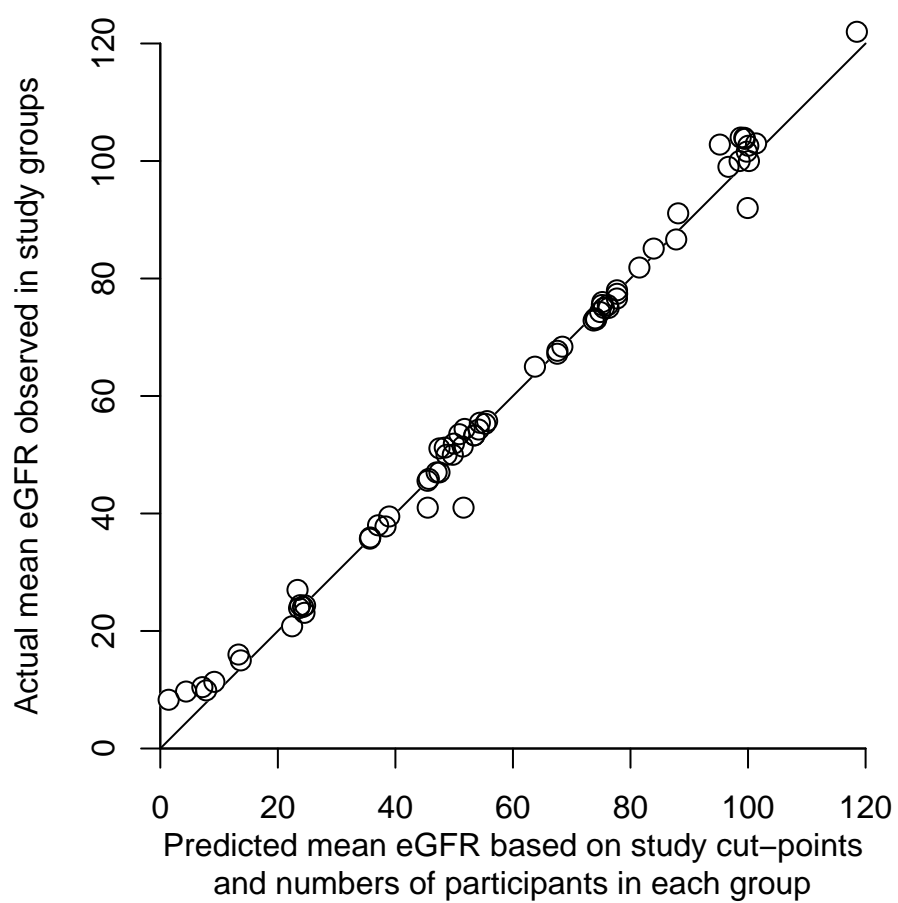

Supplement: Figure S1 — Comparison of actual vs estimated mean eGFR levels. (PDF) [file pone.0025920.s001.pdf]
