# Supplementary material for: Estimated Glomerular Filtration Rate and the Risk of Major Vascular Events and All-Cause Mortality: A Meta-Analysis
Source: PLoS One. 2011 Oct 19;6(10):e25920. doi: 10.1371/journal.pone.0025920 (PMC3198450; doi:10.1371/journal.pone.0025920)

FIGURE S2: AGE−SPECIFIC ASSOCIATION BETWEEN eGFR AND ALL−CAUSE MORTALITY IN THE VETERANS AFFAIRS STUDY

	RR (95% CI)
**Without prior CVD**	
18−44 years	1.48 (1.40, 1.57)
45−54 years	1.43 (1.39, 1.46)
55−64 years	1.39 (1.36, 1.43)
65−74 years	1.31 (1.28, 1.33)
75−84 years	1.24 (1.22, 1.27)
85−100 years	1.18 (1.13, 1.24)
**With prior CVD**	
18−44 years	1.32 (1.23, 1.42)
45−54 years	1.34 (1.31, 1.37)
55−64 years	1.32 (1.30, 1.34)
65−74 years	1.27 (1.26, 1.29)
75−84 years	1.23 (1.22, 1.24)
85−100 years	1.17 (1.15, 1.20)

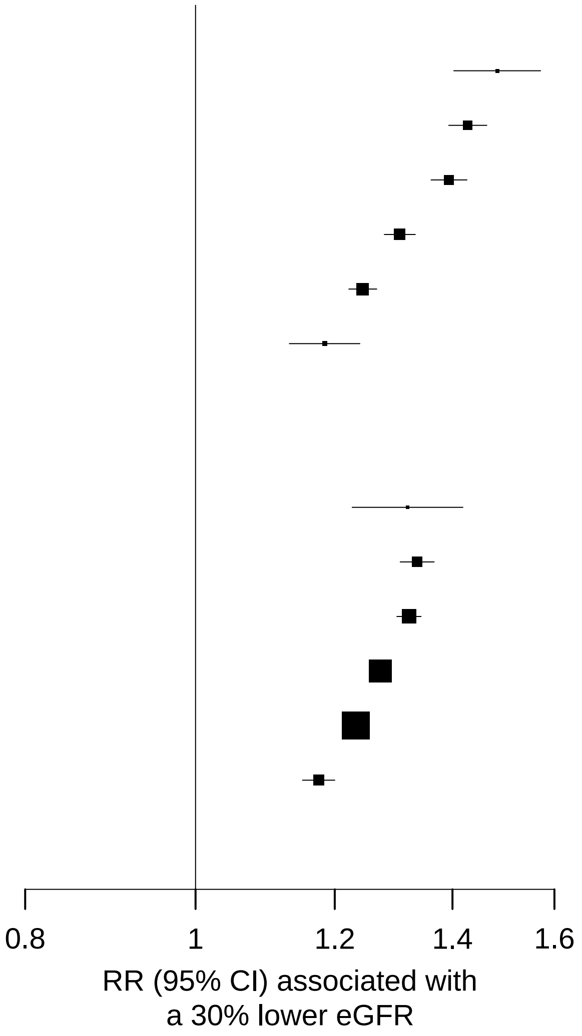

Supplement: Figure S2 — Age-specific association between eGFR and all-cause mortality in the Veterans Affairs Study. (PDF) [file pone.0025920.s002.pdf]
