# Supplementary material for: Estimated Glomerular Filtration Rate and the Risk of Major Vascular Events and All-Cause Mortality: A Meta-Analysis
Source: PLoS One. 2011 Oct 19;6(10):e25920. doi: 10.1371/journal.pone.0025920 (PMC3198450; doi:10.1371/journal.pone.0025920)
Supplement: Figure S3 — Mean follow-up eGFR level by percentile of the baseline distribution among 7697 placebo patients in the Heart Protection Study (follow-up 4-5 years later). (PDF) [file pone.0025920.s003.pdf]

FIGURE S3: MEAN FOLLOW−UP eGFR LEVEL BY PERCENTILE OF THE BASELINE DISTRIBUTION AMONG 7697 PLACEBO PATIENTS IN THE HEART PROTECTION STUDY (FOLLOW−UP 4−5 YEARS LATER)

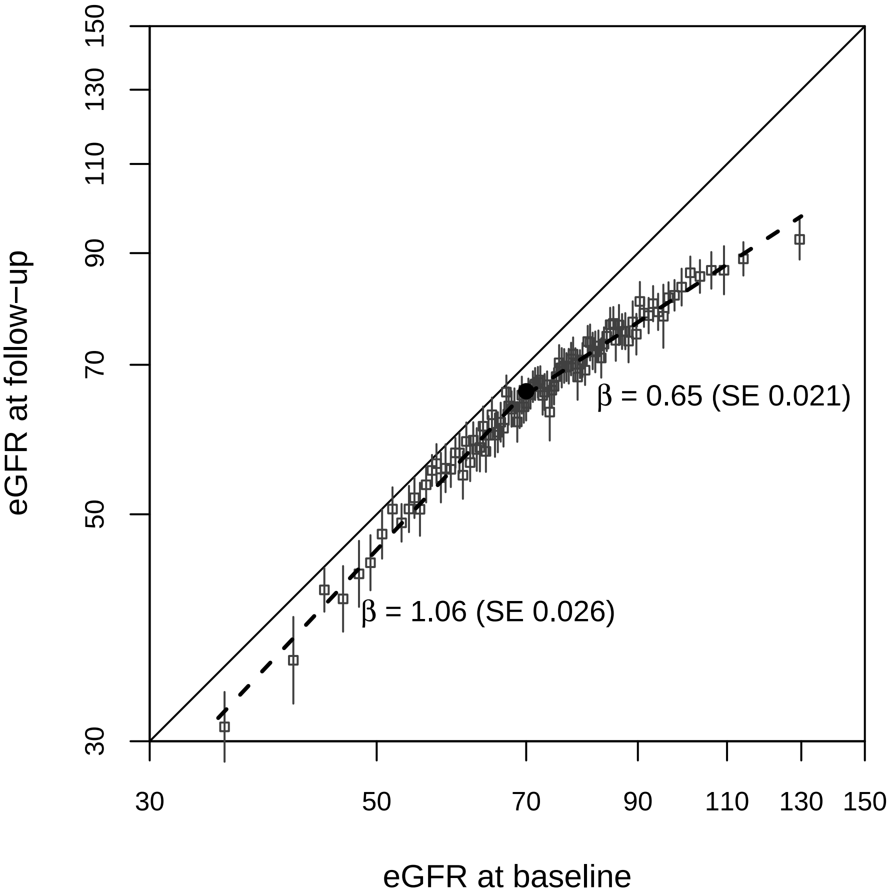

Two separate regression lines are shown, one for people whose baseline eGFR was less than 70 ml/min, and one for people whose baseline eGFR was more than 70 ml/min.
