# Supplementary material for: Estimated Glomerular Filtration Rate and the Risk of Major Vascular Events and All-Cause Mortality: A Meta-Analysis
Source: PLoS One. 2011 Oct 19;6(10):e25920. doi: 10.1371/journal.pone.0025920 (PMC3198450; doi:10.1371/journal.pone.0025920)
Supplement: Table S1 — Characteristics of included studies. (PDF) [file pone.0025920.s004.pdf]

Table S1: Characteristics of included studies

Study [reference]	n	Population	Age (years)	Men (%)	CVD (%)	FU (years	Outcomes (n)	eGFR method	Adjustments [*]
**Prospective Cohorts**									
ARIC, 2004 [w1;w2]	15006	Population sample	54	45	14	8.9	MVE (1710), Death (1325)	MDRD	BLVDSR
BIRNH, 2007 [w3]	8913	Population sample	48	53	0[†]	10[‡]	MVE (239)	MDRD	BLSW
BMES, 2005 [w4]	3074	Population sample	66	43	16	8.2	MVE[†] (224)	CG	BLVDSW
BRHS, 2006 [w5]	4029	Elderly men	69	100	19	6	MVE (304)	MDRD	BLVDSW
Cheng, 2008 [w6]	37985	Health screening	51	64	4	15	MVE (489), Death (2249)	MDRD	BLSW
Chien, 2008 [w7]	18901	Health screening	53	60	0	4.9	MVE (106), Death (500)	CG	BLDSW
Chonchol, 2007 [w8]	931	CABG	67	82	100	3.1	Death (94)	MDRD	BLDS
CHS, 2004 [w1;w2]	5616	Elderly	73	43	25	8.0	MVE (1710), Death (1880)	MDRD	BLVDSR
De Silva, 2006 [w9]	650	Stable heart failure	70	68	100	1.5[§]	Death (74)	CG	none
Framingham, 2004 [w1;w2]	6269	Population sample	54	46	12	8.9	MVE (703), Death (958)	MDRD	BLVDSR
Health ABC, 2008 [w10]	3044	Elderly	74	48	6	6[‡]	MVE (242)	MDRD	BLDSWR
Hoy, 2001 [w11]	794	Population sample	33	52	na	5.8	MVE (26), Death (63)	CG	none
JPHC, 2006 [w12]	91432	Population sample	58	33	0	10.1	MVE (1994), Death (7070)	MDRD	BLDSWP
KEEP, 2007 [w13]	37153	High risk for CKD	53	31	8	1.4	Death (191)	MDRD	BDSWRP
MONICA, 2006 [w14]	7534	Population sample	58	51	5	12.5	MVE (587), Death (1330)	MDRD	BLVDSW
NHANES II, 2002 [w15]	6354	Population sample	49	47	6	13	MVE (797), Death (1240)	MDRD	BLVDSWR
NHANES III, 2008 [w16]	14586	Population sample	44	48	8[†]	8.7[§]	MVE (887), Death (2054)	MDRD	BLVDSWR
NHS, 2004 [¶] [w17]	730	Female nurses	60	0	0	8[‡]	MVE[†] (244)	CG	BLDSWR
Nippon data 90, 2006 [w18]	7316	Population sample	52	33	0	9.6	MVE (183), Death (655)	MDRD	BLDSW
NOMAS, 2008 [w19]	3014	Population sample	69	37	24[†]	6.5	MVE (280)	CG	BLVDS
Ohasama, 2007 [w20]	1977	Health screening	63	47	5	7.8	MVE[‖] (112), Death (187)	CG	BVDSW
SMART, 2008 [w21]	3216	Stable CVD	60	66	100	3.3[§]	MVE (378), Death (337)	MDRD	BVDSW
So, 2006 [w22]	4421	Diabetes	58	43	0	3.3[§]	MVE (212), Death (111)	MDRD	BLSWP
Tsagalis, 2008 [w23]	1193	Acute stroke [**]	71	61	100	3	MVE (336), Death (~400)	MDRD	VD
Rotterdam, 2005 [w24]	4484	Population sample	70	36	0[‡‡]	8.6	MVE[‡‡] (218)	MDRD	BLVDSW
Wen, 2008 [w25]	482704	Health screening	42	48	3	7.9	MVE (3057), Death (15231)	MDRD	BLSW
WHP, 2005 [w26]	9939	Hypertension	52	62	11	9.6	MVE (573), Death (1084)	CG	BLVDSWR
Reykjavik, 2007 [¶] [w27]	18569	Population sample	55	70	0[‡‡]	20	MVE[‡‡] (2007)	MDRD	BLSW
**Retrospective cohorts**									
Amsalem, 2008 [w28]	3793	Acute heart failure [**]	73	57	100	1[‡]	Death (1072)	MDRD	BLV
Beddhu, 2002 [w29]	8600	Coronary angiogram [**]	62	68	100	3.2	MVE[‡‡] (657), Death (1320)	MDRD	BLVDS
Best, 2002 [w30]	5327	Coronary angioplasty [**]	65	71	100	2.7	Death (na)	CG	VDW [††]
Chen, 2006 [w31]	1609	Coronary angiogram [**]	61	71	100	7[§]	MVE (na), Death (382)	MDRD	BDSWR
Coceani, 2008 [w32]	1705	Coronary angiogram [**]	55	80	100	10[‡]	MVE[‡] (222), Death (341)	CG	BLVDSWP
Cox, 2008 [w33]	33386	Routine blood tests [**]	na	44	na	6[‡]	Death (6367)	MDRD	none
Dimopoulas, 2008 [w34]	1102	Congenital heart disease	36	49	100	4.1[§]	Death (103)	MDRD	none
Ezekowitz, 2004 [w35]	6427	Heart failure and CAD [**]	69	65	100	1[‡]	Death (643)	CG	LVDS
Glynn, 2007 [w36]	1272	CVD on health records	66	53	100	2.9[§]	MVE (170), Death (141)	MDRD	BLVDSW
Go, 2004 [w37]	1120295	Routine blood tests	52	45	6[†]	2.8[§]	MVE (139011), Death (51424)	MDRD	BLVDP
Goldberg, 2005 [w38]	1019	Acute MI [**]	61	88	100	1[‡]	Death (138)	MDRD	BVDS
Hillis, 2006 [w39]	2067	CABG [**]	66[§]	77	100	2.3[§]	Death (158)	MDRD	D
Holzmann, 2007 [w40]	6575	CABG	61	81	100	5[‡]	MVE[‡‡] (496), Death (628)	CG	BVD
Hwang, 2008 [w41]	35529	Elderly health screening	76	79	na	2.5	MVE (328), Death (1840)	MDRD	BLDW
Kangasniemi, 2008 [w42]	882	CABG [**]	63	75	100	10.2	MVE (308), Death (295)	MDRD	BVD
Karagiannis, 2008 [w43]	2291	Cardiac stress test	61	66	100	8	MVE[†] (417), Death (553)	CG	BLVDS
Kontos, 2005 [w44]	3074	ACS [**]	57	50	100	1[‡]	Death (259)	CG	BD

Kowalczyk, 2007 [w45]	1486	Acute MI **	58	73	100	2.5	Death (208)	MDRD	BD
Liew, 2008 [w46]	598	PVD	69	66	100	6‡	Death (195)	MDRD	BLVDS
McAlister, 2004 [w47]	754	Stable heart failure	69†	66	100	1‡	Death (203)	CG	BW
Mueller, 2004 [w48]	1400	ACS **	65	71	100	1.7	Death (82)	MDRD	BDVS
Nikolsky, 2004 [w49]	1575	PCI + Diabetes **	65	59	100	1‡	Death (125)	MDRD	BVSW
O'Hare, 2005 [w50]	5787	PVD **	69	100	100	1‡	Death (1289)	MDRD	BVDR
O'Hare, 2006 [w51]	258911	Routine blood tests	64	95	45	3.2	Death (217625)	MDRD	VDR
Smith, 2006 [w52]	44437	Acute MI **	78	50	100	1‡	Death (15020)	MDRD	BVDSR
	56652	Acute heart failure **	79	42	100	1‡	Death (21358)	MDRD	BVDSR
Smith, 2008 [w53]	118753	Acute MI **	76	51	100	10‡	Death (80707)	MDRD	BVDR
Sooklim, 2007 [w54]	1816	ACS **	65	59	100	0.9	Death (na)	MDRD	BLDS
Van Domburg, 2008 [w55]	6447	Cardiac assessment	61	74	100	7	MVE† (1093), Death (2007)	MDRD	BLVDS
Wright, 2002 [w56]	3106	Acute MI **	na	63	100	5‡	Death (na)	CG	BVD

Randomised Controlled Trials

ALLHAT, 2006 [w57]	31897	Hypertension	66	54	26†	6‡	MVE (8537)	MDRD	BLDSWR
APSIS, 2006 [w58]	808	Stable CAD	59	69	100	3.4§	MVE (69)	CG	BVD
A to Z, 2008 [w59]	4178	ACS **	60	75	100	2‡	MVE (na)	MDRD	BLVDSR
BIP, 2006 [w60]	6685	Stable CAD	60	78	100	8‡	MVE§ (287)	CG	BLVDSW
CARE, 2004 [w61]	4148	Prior MI	59	86	100	5§	MVE (592), Death (376)	CG	BLVDS
CHARM, 2006 [w62]	2680	Stable heart failure	65	67	100	2.9§	MVE (950), Death (625)	MDRD	BVDSR
DIG, 2004 [w63]	6800	Stable heart failure	64	77	100	3.1	Death (2375)	MDRD	BVDWR
HERS, 2001[w64]	2761	Stable CAD	67	0	100	4.1	MVE (682)	CG	BLVDSWR
LIPID, 2004 [w61]	8989	Prior MI	62	83	100	6.1	MVE (1555), Death (1127)	CG	BLVDS
MADIT-II, 2006 [w65]	1223	Heart failure and CAD	na	84	100	1.7	Death (216)	MDRD	VDSW
PEACE, 2006 [w66]	8280	Stable CAD	64	82	100	4.8§	MVE (696), Death (633)	MDRD	BVD
PRIME II, 2000 [w67]	1702	Stable heart failure	65	80	100	0.8	Death (432)	MDRD	BV ††
PROGRESS, 2007 [w68]	6071	Prior stroke or TIA	64	70	100	4	MVE (1058), Death (621)	CG	BDSW
SAVE, 2004 [w69]	2183	Acute MI **	59	82	100	3.5	MVE (784), Death (492)	MDRD	BVDW
SOLVED-P, 2000 [w70]	3673	Low ejection fraction	59	89	100	3.1	Death (564)	CG	BVD
SOLVED-T, 2000 [w70]	2161	Heart failure	61	81	100	3.5	Death (820)	CG	BVD
Stent-PAMI, 2003 [w71]	847	Acute MI **	60	75	100	1‡	Death (38)	CG	V ††
TAXUS-IV, 2005 [w72]	1300	Stable CAD	62	72	100	5‡	Death (26)	CG	BLVDS
TRACE, 2002 [w73]	6252	Acute MI **	68	68	100	6‡	Death (~2500)	CG	BVDS
VA-HIT, 2004 [w74]	2505	Stable CAD	64	100	100	5.1§	MVE† (491)	CG	BLVDSW
VALLIANT, 2004 [w75]	14527	Acute MI **	65	69	100	2.1§	MVE (5285), Death (3304)	MDRD	BLVDSR
VALUE, 2007 [w76]	15245	Hypertension	67	57	46	4.2	MVE (1599), Death (1659)	MDRD	V
WOSCOPS, 2004 [w61]	6588	High CVD risk	55	100	5†	4.9	MVE (514), Death (240)	CG	BLVDS

* Adjustments, in addition to age and sex, are as follows; "B", Blood pressure or history of hypertension; "L", Plasma lipids or history of hypercholesterolaemia; "V", presence or severity of vascular disease; "D", history of diabetes; "S", smoking status; "W", weight or body mass index, "R", race; "P", proteinuria; †coronary artery disease, ‡maximum, §median, ‖stroke, ¶nested case control study, ** included individuals with an acute illness at baseline, ††adjustments unclear, ‡‡myocardial infarction. "Prospective cohorts "included any non-randomised studies in which the baseline data were collected prospectively, with contemporaneous assessment of clinical measurements and laboratory blood tests using standardised methods. Studies which retrospectively extracted data from health care records was classified as a "retrospective cohort"

ACS, Acute Coronary Syndrome; ALLHAT, Antihypertensive and Lipid-Lowering Treatment to Prevent Heart Attack Trial; APSIS, Angina Prognosis in Stockholm; ARIC, Atherosclerotic Risk in Communities; BIP, Bezafibrate Infarction Prevention study; BIRNH, Belgian Inter-university Research on Nutrition and Health; BMES, Blue Mountain Eye Study; BRHS, British Regional Heart Study; CABG, Coronary Artery Bypass Graft; CAD, Coronary Artery Disease; CARE, Cholesterol And Recurrent Events; CG, Cockcroft Gault creatinine clearance (ml/min); CHARM, Candesartan in Heart Failure:Assessment of Reduction in Mortality and Morbidity; CHS, Cardiovascular Health Study; CKD, Chronic Kidney Disease; CVD, Cardiovascular Disease; DIG, Digitalis Intervention Group; FU, Follow-up; eGFR, Estimated Glomerular Filtration Rate; Health ABC, Health Aging and Body Composition; HERS, Heart and Estrogen/progestin Replacement Study; JPHC, Japan Public Health Centre-based study; KEEP, Kidney Early Evaluation Study; LIPID, Long-Term Intervention with Pravastatin in Ischaemic Disease; MADIT-II, Multicenter Automatic Defibrillator Implantation Trial-II; MDRD, Modification of Diet in Renal Disease Study eGFR (ml/min/1.73m^2); MI, Myocardial Infarction; MONICA, Monitoring Trends and Determinants on Cardiovascular Diseases in Augsburg; MVE, Major Vascular Events; na, not available; NHANES, National Health and Nutrition Estimation Survey; NHS, Nurses Health Study; NOMAS, Northern Manhattan Study; PCI, Percutaneous Coronary Intervention; PEACE, Prevention of Events with ACE inhibition; PRIME II, Second Prospective Randomized study of Ibopamine on Mortality and Efficacy; PROGRESS, Perindopril Protection Against Recurrent Stroke Study; SAVE, Survival And Ventricular Enlargement; SMART, Second Manifestations of ARTerial disease; SOLVED-P, Studies of Left Ventricular Dysfunction – Prevention; SOLVED-T, Studies of Left Ventricular Dysfunction – Treatment; Stent-PAMI, Stent Primary Angioplasty in Myocardial Infarction; TAXUS-IV, Fourth Paclitaxel-eluting stent trial; TIA, Transient Ischaemic Attack; TRACE, Trandalopril Cardiac Evaluation; VA-HIT, Veterans' Affairs High-Density Lipoprotein Intervention Trial; VALLIANT, Valsartan in Acute Myocardial Infarction Trial; VALUE, Valsartan Antihypertensive Long-term Use Evaluation; WHP, Worksite Hypertension Program; PVD, Peripheral Vascular Disease; WOSCOPS, West of Scotland Coronary Prevention Study.

Study references

w1. Weiner DE, Tighiouart H, Amin MG, Stark PC, MacLeod B et al. (2004). Chronic kidney disease as a risk factor for cardiovascular disease and all-cause mortality: a pooled analysis of community-based studies. J Am Soc Nephrol 15:1307-15.

w2. Weiner DE, Tighiouart H, Stark PC, Amin MG, MacLeod B et al. (2004) Kidney disease as a risk factor for recurrent cardiovascular disease and mortality. Am J Kidney Dis 44:198-206.

w3. Van Biesen W, De Bacquer D, Verbeke F, Delanghe J, Lameire N et al. (2007). The glomerular filtration rate in an apparently healthy population and its relation with cardiovascular mortality during 10 years. Eur Heart J 28:478-83.

w4. Leeder SR, Mitchell P, Liew G, Rochtchina E, Smith W et al. (2006). Low hemoglobin, chronic kidney disease, and risk for coronary heart disease-related death: the Blue Mountains Eye Study. J Am Soc Nephrol 17:279-84.

w5. Wannamethee SG , Shaper AG, Lowe GD, Lennon L, Rumley A et al. (2006) Renal function and cardiovascular mortality in elderly men: the role of inflammatory, procoagulant, and endothelial biomarkers. Eur Heart J 27:2975-81.

w6. Cheng TY, Wen SF, Astor BC, Tao XG, Samet JM et al. (2008). Mortality risks for all causes and cardiovascular diseases and reduced GFR in a middle-aged working population in Taiwan. Am J Kidney Dis 52:1051-60.

w7. Chien KL, Hsu HC, Lee YT, Chen MF (2008). Renal function and metabolic syndrome components on cardiovascular and all-cause mortality. Atherosclerosis 197:860-7.

w8. Chonchol MB, Aboyans V, Lacroix P, Smits G, Berl T (2007). Long-term outcomes after coronary artery bypass grafting: preoperative kidney function is prognostic. J Thorac Cardiovasc Surg 134:683-9.

w9. de Silva R, Rigby AS, Witte KK, Nikitin NP, Tin L et al. (2006). Anemia, renal dysfunction, and their interaction in patients with chronic heart failure. Am J Cardiol 98:391-8.

w10. Deo R, Fyr CL, Fried LF, Newman AB, Harris TB et al. (2008) Kidney dysfunction and fatal cardiovascular disease--an association independent of atherosclerotic events: results from the Health, Aging, and Body Composition (Health ABC) study. Am Heart J 155:62-8.

w11. Hoy WE, Wang Z, vanBuynder P, Baker PRA, Mcdonald SM et al. (2001). The natural history of renal disease in Australian Aborigines. Part 2. Albuminuria predicts natural death and renal failure. Kidney Int 60:249-256.

w12. Irie F, Iso H, Sairenchi T, Fukasawa N, Yamagishi K et al. (2006) The relationships of proteinuria, serum creatinine, glomerular filtration rate with

cardiovascular disease mortality in Japanese general population. Kidney Int. 2006;69:1264-71.

w13. McCullough PA, Jurkovitz CT, Pergola PE, McGill JB, Brown WW et al. (2007). Independent components of chronic kidney disease as a cardiovascular risk state: results from the Kidney Early Evaluation Program (KEEP). Arch Intern Med 167:1122-9.

w14. Meisinger C, Doring A, Lowel H (2006). Chronic kidney disease and risk of incident myocardial infarction and all-cause and cardiovascular disease mortality in middle-aged men and women from the general population. Eur Heart J 27:1245-50.

w15. Muntner P, He J, Hamm L, Loria C, Whelton PK (2002). Renal Insufficiency and Subsequent Death Resulting from Cardiovascular Disease in the United States. J Am Soc Nephrol 13:745.

w16. Astor BC, Hallan SI, Miller ER 3rd, Yeung E, Coresh J (2008). Glomerular filtration rate, albuminuria, and risk of cardiovascular and all-cause mortality in the US population. Am J Epidemiol 167:1226-34.

w17. Knight EL, Rimm EB, Pai JK, Rexrode KM, Cannuscio CC JE et al. (2004) Kidney dysfunction, inflammation, and coronary events: a prospective study. J Am Soc Nephrol 15:1897-903.

w18. Nakamura K, Okamura T, Hayakawa T, Kadowaki T, Kita Y et al. (2006). Chronic kidney disease is a risk factor for cardiovascular death in a community-based population in Japan: NIPPON DATA90. Circ J 70:954-9.

w19. Nickolas TL, Khatri M, Boden-Albala B, Kiryluk K, Luo X et al. (2008) The association between kidney disease and cardiovascular risk in a multiethnic cohort: findings from the Northern Manhattan Study (NOMAS). Stroke 39:2876-9.

w20. Nakayama M, Metoki H, Terawaki H, Ohkubo T, Kikuya M et al. (2007). Kidney dysfunction as a risk factor for first symptomatic stroke events in a general Japanese population--the Ohasama study. Nephrol Dial Transplant 22:1910-5.

w21. Bax L, Algra A, Mali WP, Edlinger M, Beutler JJ et al. (2008). Renal function as a risk indicator for cardiovascular events in 3216 patients with manifest arterial disease. Atherosclerosis 200:184-90.

w22. So WY, Kong AP, Ma RC, Ozaki R, Szeto CC et al. (2006). Glomerular filtration rate, cardiorenal end points, and all-cause mortality in type 2 diabetic patients. Diabetes Care 29:2046-52.

w23. Tsagalis G, Akrivos T, Alevizaki M, Manios E, Stamatellopoulos K et al. (2009). Renal dysfunction in acute stroke: an independent predictor of long-term all combined vascular events and overall mortality. Nephrol Dial Transplant 24:194-200.

w24. Brugts JJ, Knetsch AM, Mattace-Raso FU, Hofman A, Witteman JC (2005). Renal function and risk of myocardial infarction in an elderly population: the Rotterdam Study. Arch Intern Med 165:2659-65.

w25. Wen CP, Cheng TY, Tsai MK, Chang YC, Chan HT et al. (2008) All-cause mortality attributable to chronic kidney disease: a prospective cohort study based on 462 293 adults in Taiwan. Lancet 371:2173-82.

w26. Hailpern SM, Cohen HW, Alderman MH (2005). Renal dysfunction and ischemic heart disease mortality in a hypertensive population. J Hypertens 23:1809-16.

w27. Di Angelantonio E, Danesh J, Eiriksdottir G, Gudnason V. (2007) Renal function and risk of coronary heart disease in general populations: new prospective study and systematic review. PLoS Med 4:e270.

w28. Amsalem Y, Garty M, Schwartz R, Sandach A, Behar S et al. (2008). Prevalence and significance of unrecognized renal insufficiency in patients with heart failure. Eur Heart J 29:1029-36.

w29. Beddhu S, Allen-Brady K, Cheung AIK, Horne BD, Bair T et al. (2002). Impact of renal failure on the risk of myocardial infarction and death. Kidney Int 62:1776-83.

w30. Best PJM, Lennon R, Ting HH, Bell MR, Rihal CS et al. (2002). The impact of renal insufficiency on clinical outcomes in patients undergoing percutaneous coronary interventions. J Am Coll Cardiol 39:1113-9.

w31. Chen R, Kumar S, Timmis A, Feder G, Yaqoob MM et al. (2006). Comparison of the relation between renal impairment, angiographic coronary artery disease, and long-term mortality in women versus men. Am J Cardiol 97:630-2.

w32. Coceani M, Carpeggiani C, L'Abbate A (2008). Comparative prognostic value of glomerular filtration rate estimating formulas in ischaemic heart disease. Eur J Cardiovasc Prev Rehabil 15:423-7.

w33. Cox HJ, Bhandari S, Rigby AS, Kilpatrick ES (2008). Mortality at low and high estimated glomerular filtration rate values: a 'U' shaped curve. Nephron Clin Pract 110:c67-72.

w34. Dimopoulos K, Diller GP, Koltsida E, Pijuan-Domenech A, Papadopoulou SA et al. (2008). Prevalence, predictors, and prognostic value of renal dysfunction in adults with congenital heart disease. Circulation 117:2320-8.

w35. Ezekowitz J, McAlister FA, Humphries KH, Norris CM, Tonelli M et al. (2004). The association among renal insufficiency, pharmacotherapy, and outcomes in 6,427 patients with heart failure and coronary artery disease. J Am Coll Cardiol 44:1587-92.

w36. Glynn LG, Reddan D, Newell J, Hinde J, Buckley B et al. (2007). Chronic kidney disease and mortality and morbidity among patients with established

cardiovascular disease: a West of Ireland community-based cohort study. Nephrol Dial Transplant 22:2586-94.

w37. Go AS, Chertow GM, Fan D, McCulloch CE, Hsu CY (2004). Chronic kidney disease and the risks of death, cardiovascular events, and hospitalization. N Engl J Med 351:1296-305.

w38. Goldberg A, Hammerman H, Petcherski S, Zdorovyak A, Yalonetsky S et al. (2005). Inhospital and 1-year mortality of patients who develop worsening renal function following acute ST-elevation myocardial infarction. Am Heart J 150:330-7.

w39. Hillis GS, Croal BL, Buchan KG, El-Shafei H, Gibson G et al. (2006). Renal function and outcome from coronary artery bypass grafting: impact on mortality after a 2.3-year follow-up. Circulation 113:1056-62.

w40. Holzmann MJ, Hammar N, Ahnve S, Nordqvist T, Pehrsson K et al. (2007). Ivert T. Renal insufficiency and long-term mortality and incidence of myocardial infarction in patients undergoing coronary artery bypass grafting. Eur Heart J 28:865-71.

w41. Hwang SJ, Lin MY, Chen HC, Hwang SC, Yang WC et al. (2008). Increased risk of mortality in the elderly population with late-stage chronic kidney disease: a cohort study in Taiwan. Nephrol Dial Transplant 23:3192-8.

w42. Kangasniemi OP, Mahar MA, Rasinaho E, Satomaa A, Tiozzo V et al. (2008). Impact of estimated glomerular filtration rate on the 15-year outcome after coronary artery bypass surgery. Eur J Cardiothorac Surg 33:198-202.

w43. Karagiannis SE , Feringa HH, Elhendy A, van Domburg R, Chonchol M et al. (2008). Prognostic significance of renal function in patients undergoing dobutamine stress echocardiography. Nephrol Dial Transplant 23:601-7.

w44. Kontos MC, Garg R, Anderson FP, Tatum JL, Ornato JP et al. (2005). Predictive power of ejection fraction and renal failure in patients admitted for chest pain without ST elevation in the troponin era. Am Heart J 150:666-73.

w45. Kowalczyk J, Lenarczyk R, Kowalski O, Sredniawa B, Musialik-Lydka A et al. (2007). Different types of renal dysfunction in patients with acute myocardial infarction treated with percutaneous coronary intervention. J Interv Cardiol 20:143-52.

w46. Liew YP, Bartholomew JR, Demirjian S, Michaels J, Schreiber MJ Jr (2008). Combined effect of chronic kidney disease and peripheral arterial disease on all-cause mortality in a high-risk population. Clin J Am Soc Nephrol 3:1084-9.

w47. McAlister FA, Ezekowitz J, Tonelli M, Armstrong PW (2004). Renal insufficiency and heart failure: prognostic and therapeutic implications from a prospective cohort study. Circulation 109:1004-9.

w48. Mueller C, Neumann FJ, Perruchoud AP, Buettner HJ (2004). Renal function and long term mortality after unstable angina/non-ST segment elevation

myocardial infarction treated very early and predominantly with percutaneous coronary intervention. Heart 90:902-7.

w49. Nikolsky E, Mehran R, Turcot D, Aymong ED, Mintz GS et al. (2004). Impact of chronic kidney disease on prognosis of patients with diabetes mellitus treated with percutaneous coronary intervention. Am J Cardiol 94:300-5.

w50. O'Hare AM, Bertenthal D, Shlipak MG, Sen S, Chren MM (2005). Impact of renal insufficiency on mortality in advanced lower extremity peripheral arterial disease. J Am Soc Nephrol 16:514-9.

w51. O'Hare AM, Bertenthal D, Covinsky KE, Landefeld CS, Sen S et al. (2006). Mortality risk stratification in chronic kidney disease: one size for all ages? J Am Soc Nephrol 17:846-53.

w52. Smith GL, Shlipak MG, Havranek EP, Foody JM, Masoudi FA et al. (2006). Serum urea nitrogen, creatinine, and estimators of renal function: mortality in older patients with cardiovascular disease. Arch Intern Med 166:1134-42.

w53. Smith GL, Masoudi FA, Shlipak MG, Krumholz HM, Parikh CR (2008). Renal impairment predicts long-term mortality risk after acute myocardial infarction. J Am Soc Nephrol 19:141-50.

w54. Sooklim K, Srimahachota S, Boonyaratavej S, Kanjanavanit R, Siriviwattanakul N et al. (2007). Renal dysfunction as an independent predictor of total mortality after acute coronary syndrome: the Thai ACS Registry. J Med Assoc Thai 90 Suppl 1:32-40.

w55. van Domburg RT, Hoeks SE, Welten GM, Chonchol M, Elhendy A et al. (2008). Renal insufficiency and mortality in patients with known or suspected coronary artery disease. J Am Soc Nephrol 19:158-63.

w56. Wright R, Scott R, Guy S, Herzog CA, Albright RC at al. (2002) Acute Myocardial Infarction and Renal Dysfunction: A High-Risk Combination. Ann Intern Med 137:563-570.

w57. Rahman M, Pressel S, Davis BR, Nwachuku C, Wright JT Jr et al. (2006) Cardiovascular Outcomes in High-Risk Hypertensive Patients Stratified by Baseline Glomerular Filtration Rate. Ann Intern Med 144:172-80.

w58. Almquist T, Forslund L, Rehnqvist N, Hjemdahl P (2006). Prognostic implications of renal dysfunction in patients with stable angina pectoris. J Intern Med 260:537-44.

w59. Mielniczuk LM, Pfeffer MA, Lewis EF, Blazing MA, de Lemos JA et al. (2008) Estimated glomerular filtration rate, inflammation, and cardiovascular events after an acute coronary syndrome. Am Heart J 155:725-31.

w60. Koren-Morag N, Goldbourt U, Tanne D (2006). Renal dysfunction and risk of ischemic stroke or TIA in patients with cardiovascular disease. Neurology 67:224-8.

w61. Tonelli M, Isles C, Curhan GC, Tonkin A, Pfeffer MA et al. (2004) Effect of pravastatin on cardiovascular events in people with chronic kidney disease. Circulation 110:1557-63.

w62. Hillege HL, Nitsch D, Pfeffer MA, Swedberg K, McMurray JJ et al. (2006). Renal function as a predictor of outcome in a broad spectrum of patients with heart failure. Circulation 113:671-8.

w63. Shlipak MG, Smith GL, Rathore SS, Massie BM, Krumholz HM (2004). Renal function, digoxin therapy, and heart failure outcomes: evidence from the digoxin intervention group trial. J Am Soc Nephrol 15:2195-203.

w64. Shlipak MG, Simon JA, Grady D, Lin F, Wenger NK et al. (2001). Heart and Estrogen/progestin Replacement Study (HERS) Investigators. Renal insufficiency and cardiovascular events in postmenopausal women with coronary heart disease. J Am Coll Cardiol 38:705-11.

w65. Goldenberg I, Moss AJ, McNitt S, Zareba W, Andrews ML et al. (2006). Relations among renal function, risk of sudden cardiac death, and benefit of the implanted cardiac defibrillator in patients with ischemic left ventricular dysfunction. Am J Cardiol 98:485-90.

w66. Solomon SD, Rice MM, A Jablonski K, Jose P, Domanski M et al. (2006). Renal function and effectiveness of angiotensin-converting enzyme inhibitor therapy in patients with chronic stable coronary disease in the Prevention of Events with ACE inhibition (PEACE) trial. Circulation 114:26-31.

w67. Hillege HL, Girbes ARJ, de Kam PJ, Boomsma F, de Zeeuw D et al. (2000). Renal Function, Neurohormonal Activation, and Survival in Patients With Chronic Heart Failure. Circulation 102:203-10.

w68. Perkovic V, Ninomiya T, Arima H, Gallagher M, Jardine M et al. (2007). Chronic kidney disease, cardiovascular events, and the effects of perindopril-based blood pressure lowering: data from the PROGRESS study. J Am Soc Nephrol 18:2766-72.

w69. Tokmakova MP, Skali H, Kenchaiah S, Braunwald E, Rouleau JL et al. (2004). Chronic kidney disease, cardiovascular risk, and response to angiotensin-converting enzyme inhibition after myocardial infarction: the Survival And Ventricular Enlargement (SAVE) study. Circulation 110:3667-73.

w70. Dries DL, Exner DV, Domanski MJ, Greenberg B, Stevenson LW (2000). The prognostic implications of renal insufficiency in asymptomatic and symptomatic patients with left ventricular systolic dysfunction. J Am Coll Cardiol 35:681-9.

w71. Dixon SR, O'Neill WW, Sadeghi HM, Stone GW, Brodie B et al. (2003). Usefulness of creatinine clearance in predicting early and late death after primary angioplasty for acute myocardial infarction. Am J Cardiol 91:1454-7.

w72. Halkin A, Mehran R, Casey CW, Gordon P, Matthews R et al. (2005). Impact of moderate renal insufficiency on restenosis and adverse clinical events after

paclitaxel-eluting and bare metal stent implantation: results from the TAXUS-IV Trial. Am Heart J 150:1163-70.

w73. Sorensen CR, Brendorp B, Rask-Madsen C, Kober L, Kjoller E et al. (2002). The prognostic importance of creatinine clearance after acute myocardial infarction. Eur Heart J 23:948-52.

w74. Tonelli M, Collins D, Robins S, Bloomfield H, Curhan GC (2004). Gemfibrozil for secondary prevention of cardiovascular events in mild to moderate chronic renal insufficiency. Kidney Int 66:1123-30.

w75. Anavekar NS, McMurray JJ, Velazquez EJ, Solomon SD, Kober L et al. (2004). Relation between renal dysfunction and cardiovascular outcomes after myocardial infarction. N Engl J Med 351:1285-95.

w76. Ruilope LM, Zanchetti A, Julius S, McInnes GT, Segura J et al. (2007). Prediction of cardiovascular outcome by estimated glomerular filtration rate and estimated creatinine clearance in the high-risk hypertension population of the VALUE trial. J Hypertens 25:1473-9.
