# Supplementary material for: Estimated Glomerular Filtration Rate and the Risk of Major Vascular Events and All-Cause Mortality: A Meta-Analysis
Source: PLoS One. 2011 Oct 19;6(10):e25920. doi: 10.1371/journal.pone.0025920 (PMC3198450; doi:10.1371/journal.pone.0025920)
Supplement: Appendix S1 — PubMed search conducted on 1st September 2008. (PDF) [file pone.0025920.s005.pdf]

Appendix S1: PubMed search conducted on 1st September 2008

Step		Citations
1	"cardiovascular diseases/epidemiology"[MeSH Terms] OR "heart diseases/epidemiology"[MeSH Terms] OR "coronary disease/epidemiology"[MeSH Terms] OR "myocardial infarction/epidemiology"[MeSH Terms] OR "cerebrovascular disorders/epidemiology"[MeSH Terms] OR "death/epidemiology"[MeSH Terms] OR "vascular diseases/epidemiology"[MeSH Terms] OR "cardiovascular abnormalities/epidemiology"[MeSH Terms]	149541
2	"renal failure" OR "renal impairment" OR "renal disease" OR "kidney disease" OR "chronic kidney failure" OR "chronic kidney insufficiency" OR "glomerular filtration" OR "glomerular filtration rate" OR "kidney function test" OR "kidney function tests" OR "kidney function" OR "kidney function creatinine clearance" OR "kidney function serum creatinine" OR "kidney clearance" OR "kidney clearance rates" OR "kidney clearance tests" OR "kidney clearances"	165524
3	#1 AND #2	5277
4	"cardiovascular diseases " OR "heart diseases" OR "coronary disease " OR "myocardial infarction " OR "cerebrovascular disorders " OR "death " OR "vascular diseases " OR "cardiovascular abnormalities "	765430
5	"renal failure" OR "renal impairment" OR "renal disease" OR "kidney disease" OR "chronic kidney failure" OR "chronic kidney insufficiency" OR "glomerular filtration" OR "glomerular filtration rate" OR "kidney function test" OR "kidney function tests" OR "kidney function" OR "kidney function creatinine clearance" OR "kidney function serum creatinine" OR "kidney clearance" OR "kidney clearance rates" OR "kidney clearance tests" OR "kidney clearances" OR "creatinine/blood" OR "kidney/abnormalities" OR "kidney/epidemiology" OR "kidney/physiology" OR "kidney/physiopathology"	204568
6	"prognosis" OR "risk factors" OR "survival analysis" OR "comparative study" OR "prospective studies" OR "cause of death" OR "cohort study" OR "epidemiology"	2850498
7	#4 AND #5 AND #6	9544
8	#3 OR #7	11981
