# Supplementary material for: Estimated Glomerular Filtration Rate and the Risk of Major Vascular Events and All-Cause Mortality: A Meta-Analysis
Source: PLoS One. 2011 Oct 19;6(10):e25920. doi: 10.1371/journal.pone.0025920 (PMC3198450; doi:10.1371/journal.pone.0025920)
Supplement: Appendix S2 — Statistical appendix. (PDF) [file pone.0025920.s006.pdf]

Appendix S2: Statistical appendix

Estimating the mean eGFR level in groups defined by "cutoff" values

If *X* has a normal distribution with mean μ and standard deviation σ, then it follows that $Z = (X - \mu)/\sigma$ has a normal distribution with mean 0 and standard deviation 1. Now let X' be the same as X but constrained to lie between two cutoff values a and b. The expected value of X' is equal to $E(X \mid a < X < b) = \mu + \sigma E(Z \mid \frac{a-\mu}{\sigma} < Z < \frac{b-\mu}{\sigma})$. The probability density function of Z conditional on Z taking a value between $\frac{a-\mu}{\sigma}$ and $\frac{b-\mu}{\sigma}$ is:

$$\Pr\left(Z < z \mid \frac{(a-\mu)}{\sigma} < Z < \frac{(b-\mu)}{\sigma}\right) = \frac{1}{\Phi(\frac{(b-\mu)}{\sigma}) - \Phi(\frac{(a-\mu)}{\sigma})} \int_{\frac{(a-\mu)}{\sigma}}^{z} \phi_W \, dw$$

where ϕ is the standard normal density function and Φ is the standard normal cumulative distribution function. Therefore, the expected value of X' is equal to:

$$\mu + \frac{\sigma}{\Phi(\frac{b-\mu}{\sigma}) - \Phi(\frac{a-\mu}{\sigma})} \int_{\frac{a-\mu}{\sigma}}^{\frac{b-\mu}{\sigma}} w\phi_W \, dw$$

$$= \mu + \frac{\sigma}{\Phi(\frac{b-\mu}{\sigma}) - \Phi(\frac{a-\mu}{\sigma})} \cdot \frac{1}{\sqrt{2\pi}} \left(e^{-\frac{1}{2}\left(\frac{a-\mu}{\sigma}\right)^2} - e^{-\frac{1}{2}\left(\frac{b-\mu}{\sigma}\right)^2} \right) \quad (1)$$

However, in studies in which the overall mean and standard deviation were not stated, these were first estimated from the known proportions of the population falling within each exposure group. For instance, if a study of *N* participants reported three exposure groups: eGFR $< a$; $\geq a, < b$ and $\geq b$ ml/min/1.73m^2; with n_1, n_2 *and* n_3 participants falling, respectively, into each category, then, under the normality assumption:

$$\frac{a - \hat{\mu}}{\hat{\sigma}} = \Phi^{-1}(p_1)$$

and

$$\frac{b-\hat{\mu}}{\hat{\sigma}} = \Phi^{-1}(p_2)$$

where $p_1 = n_1/N$ and $p_2 = (n_1 + n_2)/N$, and so:

$$\hat{\sigma} = \frac{b-a}{\Phi^{-1}(p_2) - \Phi^{-1}(p_1)} \tag{2}$$

and

$$\hat{\mu} = a - \hat{\sigma}.\Phi^{-1}(p_1) \tag{3}$$

For studies in which more than 3 exposure groups were used however (and hence any two cutoff values could potentially be used to calculate the mean and standard deviation of the overall distribution), the mean eGFR in each exposure group was calculated from estimates of μ and σ derived from the nearest two cutoff levels (i.e. either the cutoff levels actually defining the group or, if the group is one of the tails of the distribution, from the two nearest cutoff levels).
